# Clinical practice guidelines for the treatment and management of diabetic macular oedema: a systematic review

**DOI:** 10.1038/s41433-025-04043-2

**Published:** 2025-10-01

**Authors:** Mark McAllister, Stella Ko, Komal Bawa, Elizabeth Mearns, David Tabano, Amanda Martinez, Nancy Faux, Shriji Patel, Galin Spicer, Jennifer I. Lim

**Affiliations:** 1 Department of Ophthalmology and Visual Sciences, University of Illinois Chicago, Chicago, IL USA; 2 Genentech Inc, South San Francisco, CA USA; 3 College of Medicine at The Ohio State University, Columbus, OH USA

**Keywords:** Diagnosis, Eye diseases

## Abstract

**Background/objectives:**

To assess geographically global clinical practice guidelines (CPGs) for diabetic macular oedema (DMO) management.

**Methods:**

A systematic literature review (SLR) of CPGs for DMO management was conducted using Embase and MEDLINE databases, Guideline Central, Health Technology Assessment bodies, professional ophthalmology associations, and backwards citation tracking. CPGs published between January 2010–October 2023 were included and independently assessed by four reviewers and one adjudicator using the Appraisal of Guidelines for Research and Evaluation (AGREE II) instrument. CPGs were qualitatively assessed for anatomical measurement (optical coherence tomography [OCT]) and visual acuity (VA) recommendations. PROSPERO identification: CRD42023473223.

**Results:**

14/147 identified CPGs were included. Overall AGREE II scores were 49–91 (mean [SD] = 67 [11]). Strongest domains were Scope and Purpose (85 [10.9]), Clarity of Presentation (87 [18.7]), and Editorial Independence (91 [13.7]). Stakeholder Involvement (57 [8.3]), Applicability (54 [19.4]), and Rigor of Development (41 [19.0]) scored lowest. 13/14 CPGs were “Recommended” or “Recommended with Modifications”. All CPGs recommended OCT for initial diagnosis. 3/14 CPGs did not recommend VA or considered VA optional. For initial disease management, 11/14 CPGs recommended OCT. One considered OCT optional. VA was recommended by 9/14 CPGs for initial management. All CPGs recommended using VA and OCT for disease monitoring while on anti-vascular endothelial growth factor therapy. 12/14 CPGs recommended using OCT to measure anti-VEGF response to adjust treatment interval.

**Conclusion:**

CPGs were aligned regarding the importance of OCT in DMO management. More rigorous methods, applicability in resource-constrained systems, and patient perspectives will improve CPG trustworthiness and transparency.

## Introduction

Diabetic retinopathy (DR) is a severe complication of diabetes that is characterised by increased retinal vascular permeability, oedema, ischaemia, and neovascularisation that results in visual impairment and blindness [1, 2]. Diabetic macular oedema (DMO) can develop at any point in a patient with DR but occurs most often with increasing DR severity (e.g., proliferative DR) [1, 2]. As of 2022, the pooled global prevalence of DMO in patients diagnosed with diabetes was around 5.47% (95% confidence interval [CI], 3.66–7.62%) [3] and is expected to continue increasing [4]. An increasing number of people will incur a detriment on their quality of life, as left untreated [5], DMO can lead to permanent vision loss [6].

Anyone diagnosed with type 1 or 2 diabetes is at risk of diabetic eye disease. DR and DMO are often suspected when these patients present with vision loss [1]. A formal diagnosis is later confirmed through a detailed ocular examination, including a visual acuity (VA) assessment, dilated eye examination, optical coherence tomography (OCT), fluorescein angiography, and tonometry [2]. In addition, retina specialists leverage OCT to support both diagnosis and disease management in DR and DMO [7], thereby providing both subjective and objective evidence of retinal thickening and oedema [8–10]. OCT is a non-invasive tool that measures the central subfield thickness of the retina by using cross-sectional images [11].

Unlike other therapeutic areas with authoritative prescribing and disease management guidelines (e.g., National Comprehensive Cancer Network guidelines for oncologic conditions or American College of Rheumatology for immunological conditions), ophthalmology recommendations within clinical practice guidelines (CPGs) are variable, most notably with regard to the latitude that retina specialists are given to base their management decisions. The *International Council of Ophthalmology* updated guidelines for diabetic eye care in 2018 to specifically include use of OCT in the management of DMO, given its extensive use in clinical practice [12]. Among patients with DMO, OCT parameters are associated with various prognostic outcomes and may determine clinical response to interventions.

Over the past two decades, the disease landscape has been transformed by the advent of treatment mainstays, such as intravitreal treatments (e.g., anti-vascular endothelial growth factor [anti-VEGF] and steroids), macular laser photocoagulation, and surgical interventions [13]. However, pharmacological management has become the primary treatment modality for DMO [14]. Health care professionals rely on CPGs for recommendations on diagnosis and management strategies, including when to administer a patient’s “next” anti-VEGF dose based on OCT imaging, which measures anatomical response, including subfoveal thickness and fluid management (i.e., “treat to drying”). Thus, there are variations in quality among CPG recommendations for DMO diagnosis, disease monitoring, and anti-VEGF prescribing practice, depending on their use of OCT for disease management decision-making. Moreover, payers often anchor to the primary endpoints from pivotal clinical trials when determining access to medications, yet most DMO randomised controlled trials report VA as the primary endpoint, which is considered less objective than OCT for disease monitoring in real-world clinical practice [15]. As such, there is a disconnect between real-world practice and randomised controlled trials, as anti-VEGF agents are approved by VA primary endpoints but are often prescribed based on anatomical secondary endpoints.

Presently, there is a lack of standardised best practices for the management of DMO. Many retina specialists and ophthalmologists in academic practice are likely more knowledgeable about the most up-to-date literature; however, in more resource-constrained settings, treatment may be more variable. This could lead to potential health disparity issues where the quality of care patients are receiving might be different depending on the care settings. To address these issues, we conducted a systematic literature review (SLR) of published CPGs for the management of adults with DMO. We aimed to assess the quality of the CPG, and strength of recommendations for OCT and VA measurements to help provide direction on improvements for the development of future CPGs.

## Methods

### Eligibility criteria for considering studies for this review

#### Search methods for identifying studies

Search strategies were developed using a combination of controlled vocabulary and keywords, excluding studies before 2010. Searches were conducted in MEDLINE and Embase databases to identify CPGs published between 1 January 2010 and 20 October 2023 that were limited to English-language publications. A full list of the search terms is detailed in Supplementary Table S1. Manual backwards citation tracking of references from included CPGs and review articles was performed to identify additional relevant CPGs in the grey literature. Searches were also performed in various websites (e.g., EyeWiki, Guideline Central, Google Scholar, and ophthalmology and retina society portals [e.g., American Academy of Ophthalmology, International Council of Ophthalmology, American Society of Retina Specialists, The Royal College of Ophthalmologists, and European Society of Retina Specialists]).

#### Eligibility criteria for considering studies for this review

Eligible CPGs from any country that recommended management strategies for adult patients with DMO were included. Country adaptations of CPGs or those limited to a single anti-VEGF agent were considered outside the scope of this SLR and were excluded.

### Study selection

Two reviewers independently screened titles and abstracts (K.B. and E.M.) to identify publications that met the inclusion criteria. Discrepancies were resolved between the two reviewers through discussion. If an agreement was not reached, a third reviewer (S.K.) resolved any disagreements. The full-text publication review followed the same process.

### Data collection and risk of bias assessment

A standardised extraction template in Google Forms^®^ was developed to capture and present key evidence from each CPG included. Two independent reviewers (S.K. and M.M.) extracted the following data from each CPG: author, year of publication, professional society, country/region, funding, medical specialty of CPG development committee, and recommendations regarding the use of OCT and VA in the diagnosis, treatment, and management of DMO. Consensus methods utilised by the CPG were also captured (e.g., Delphi approach, informal consensus, GRADE). A Delphi survey method was defined as anonymity among the experts who developed the CPG, which would prevent group members from conforming with the opinions of others [16].

### Data synthesis, reporting quality, and analysis

The reporting quality of included CPGs was independently assessed by four reviewers (K.B., S.K., M.M., A.M.) using the validated Appraisal of Guidelines for Research and Evaluation II (AGREE II) instrument. AGREE II is a tool developed to assess the quality and methodological rigour of published CPGs [17–19]. It is composed of 23 items across quality domains: Scope and Purpose, Stakeholder Involvement, Rigour of Development, Clarity of Presentation, Applicability, and Editorial Independence (Supplementary Table S2). All reviewers were trained on the AGREE II tool before completing the appraisal. All reviewers rated each item on a 7-point scale from 1 (strongly disagree) to 7 (strongly agree). After each reviewer independently scored the CPG, domain percentages were calculated using the AGREE II methodology (domain scores were calculated by summing up all the scores of the individual items in a domain and by scaling the total as a percentage of the maximum possible score for that domain) [19]. Since the AGREE II tool does not provide a threshold percentage score to dictate the level of quality in each domain, the reviewers instituted a threshold of 0–30% for low quality, 31–70% for moderate, and ≥71% high quality.

An overall assessment of the CPG included two items: overall quality rating of the CPG and whether the CPG would be recommended for use in practice using a 3-point scale (Recommend, Recommend with Modifications, Do Not Recommend). Overall quality rating percentages were calculated by summing up scores across all four reviewers and scaling across the total maximum possible score across all domains. For the overall recommendation for use in clinical practice, independent reviewers used their judgement as to the quality of the CPG, taking into account the criteria in the AGREE II assessment process. Results of the SLR were summarised qualitatively using narrative synthesis. If an agreement was not reached, a third reviewer (E.M. or D.T.) resolved any disagreements.

This SLR was conducted in accordance with the guidelines established in the Cochrane Handbook for Systematic Reviews of Interventions [20] and this publication followed the methodology of the Preferred Reporting Items for Systematic Reviews and Meta-analyses reporting guidelines [21]. PROSPERO number for this SLR is CRD42023473223.

## Results

### Search results

**Fig. 1:**
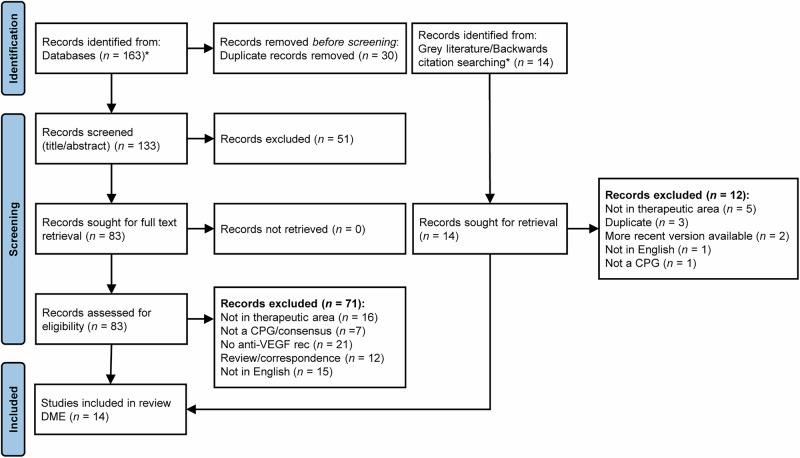
PRISMA flow diagram including database searches and grey literature sources [21]. ^a^ Initial literature search identified publications in DMO, nAMD, DR, and RVO. CPG clinical practice guideline, DMO diabetic macular oedema; VEGF vascular endothelial growth factor. For more information, visit: http://www.prisma-statement.org/.

The CPG selection process is summarised in (Fig. 1). The electronic searches yielded 133 unique records for title and abstract screening after removing 30 duplicates. A total of 14 records were identified and retrieved from the grey literature and backwards citation searches. After applying Population, Intervention, Comparison, Outcomes, and Study (PICOS) criteria, a total of 83 records were reviewed in full text, of which 14 CPGs were included. PICOS registered on PROSPERO are broadly similar to those presented here; however, there are some data extraction criteria that were combined because of a lack of granularity in some of the retrieved CPGs.

### Description of CPGs

Table 1 presents characteristics of the 14 CPGs included [22–35]. All CPGs were published between 2017 and 2023, with 11 of the 14 published more recently since 2020. Most guidelines (9/14) were developed under the purview of professional societies or health ministries, with the remainder developed by expert consensus. Retina specialists were identified in 10 of the CPGs as the development experts, whereas the others included a combination of ophthalmologists and other experts, such as endocrinologists, surgeons, and public health specialists. Regional representation was highest in the Asia-Pacific region (*n* = 6), followed by Europe (*n* = 4), the Middle East (*n* = 2), and North America (*n* = 2). Only four CPGs used an anonymous Delphi method to reach consensus and only six included a literature review to amass evidence on which to base their clinical recommendations.

**Table 1:** Characteristics of CPGs.

First author/year	Institution/professional group	Region/country	CPG development experts	Consensus method	Methods included a literature review (Y/N)
Al Qahtani [22]	Saudi Retina Group	Saudi Arabia	Ophthalmologists	Consensus	N
Al Qassimi [23]	Emirates Society of Ophthalmology	United Arab Emirates	Ophthalmologists	Consensus	N
Amoaku [24]	Royal College of Ophthalmology	United Kingdom	Retina specialists, diabetologists, vitreoretinal surgeons, public health professionals	Consensus	Y
Bakri [25]	American Society of Retina Specialists	USA	Retina specialists	Consensus	N
Chen [26]	N/R	Taiwan	Ophthalmologists	Consensus	N
Cheung [27]	N/R	Asia^a^	Retina specialists	Consensus	Y
Chhablani 2020 [28]	N/R	Asia-Pacific^b^	Retina specialists	Delphi	N
Fernandez-Vigo [29]	N/R	Spain	Retina specialists	Consensus	Y
Flaxel [30]	American Academy of Ophthalmology	USA	Retina specialists	SIGN, GRADE	Y
Gilbert [31]	Indian Institute of Public Health	India	Ophthalmologists, community physicians, policymakers, and public health professionals	Delphi	Y
Giridhar [32]	All India Ophthalmological Society and Vitreo-Retinal Society of India	India	Retina specialists	Delphi	N
Ngah [33]	Malaysia Retina Group	Malaysia	Retina specialists	Consensus	N
Schmidt-Erfurth [34]	European Society of Retina Specialists	Europe	Retina specialists	Delphi	N
Udaondo [35]	N/R	Spain	Retina specialists	Consensus	Y

*CPG* clinical practice guideline, *GRADE* Grading of Recommendations Assessment, Development and Evaluation, *N/R* not reported, *SIGN* Scottish Intercollegiate Guideline Network.

^a^Asian countries included were Singapore, South Korea, Taiwan, Hong Kong, Malaysia, Thailand, Indonesia, and the Philippines.

^b^Asian countries included were Singapore, Malaysia, Philippines, India, and Vietnam.

### Recommendations for OCT and VA assessments

**Fig. 2:**
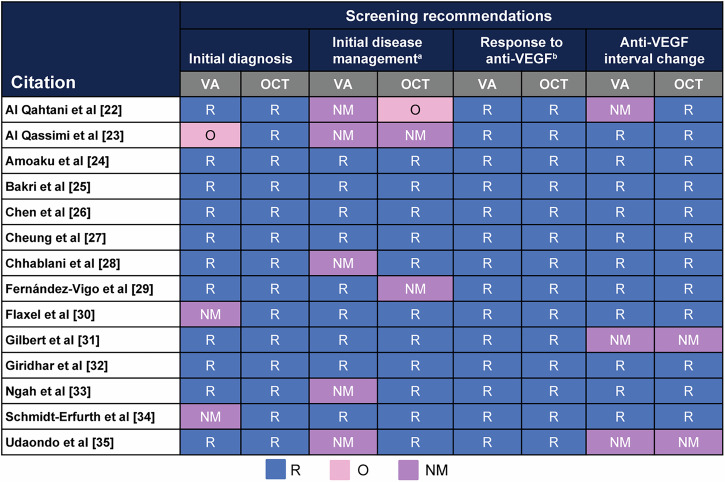
Screening recommendations for Adults with DMO. ^a^ Management of initial pharmacologic intervention. ^b^Screening to assess disease progression moderate quality (31–70%); red = low-quality (<30%). DMO diabetic macular oedema, NM not mentioned, O optional, OCT optical coherence tomography, R recommend, VA visual acuity, VEGF vascular endothelial growth factor.

All CPGs recommended leveraging OCT for initial DMO diagnosis. Most CPGs (11/14) also recommended VA for initial DMO diagnosis (Fig. 2). The OCT measurement was the basis of the initial disease management recommendation in 11/14 CPGs, whereas two did not recommend OCT, and one recommended OCT as optional. VA was recommended less frequently (9/14) with five guidelines providing no recommendation for VA.

All 14 CPGs provided guidance on assessing anti-VEGF response and recommended using both OCT and VA to monitor patients for progression. Furthermore, 12/14 CPGs recommended OCT to assess the potential for modifying the anti-VEGF treatment interval. Similarly, 11 CPGs recommended leveraging VA for modifying the anti-VEGF treatment interval. Among the CPGs assessed, none provided recommendations for which specific anti-VEGF therapy to choose. Although not the focus of this review, the use of steroids in the management of DMO was noted to be more variable than anti-VEGF therapy.

### Quality appraisal of CPGs using AGREE II

**Fig. 3:**
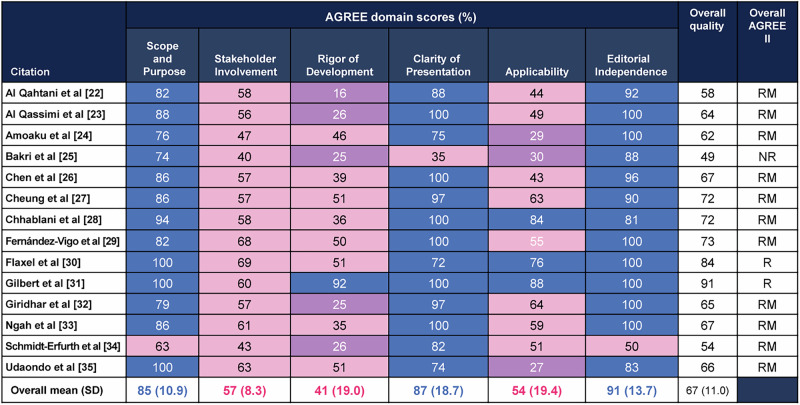
Standardised scores for each domain using the AGREE II instrument. Domain scores were rounded. Blue = high quality (>71%); pink = moderate quality (31–70%); purple = low quality (<30%). *AREE II* Appraisal of Guidelines for Research and Evaluation II, *CPG* clinical practice guideline, *NR* do not recommend, *R* recommend, RM recommend with modifications.

Figure 3 highlights the AGREE II quality assessment scores of the CPGs. The overall mean scores ranged from 41% to 91%, with the Scope and Purpose (85; standard deviation [SD], 10.9), Clarity of Presentation (87; SD, 18.7), and Editorial Independence (91; SD, 13.7) domains having the highest scores. The Rigor of Development was the weakest domain with the lowest overall mean score (41; SD, 19.0) and five of the 14 CPGs scoring a low overall quality (<30%). “Rigor of Development” was the lowest domain score across all domains for all CPGs except the Indian Institute of Public Health [31]. The main contributing factors to these low domain scores for “Rigor of Development” came from the majority of CPGs failing to employ or report conducting a systematic literature review or pragmatic methodology for identifying literature that informed the recommendations (Table 1). Additionally, all but one CPG [31] failed to report a procedure for updating their guideline.

Notably, although each CPG included individuals from relevant professional groups who would treat patients with DMO, CPGs consistently failed to include the “views and preferences of the target population”, which was reflected in the low scores for stakeholder involvement. Similarly, applicability information, such as the facilitators/barriers to application or the resource implications of applying the recommendations, was often absent in the CPGs.

When evaluating the overall quality of the CPGs analysed, most of the CPGs could be “Recommended with Modifications”; two were fully “Recommend”, and only one was “Do Not Recommend”. Overall, the four independent assessors noted that they would “Recommend” or “Recommend with Modifications” 13 of the 14 CPGs assessed. Modifications suggested for the CPGs that were categorised as “Recommended with Modifications” included a more explicit link between the literature and recommendations, to improve clarity in recommendations, and potential to update the CPG given the changing disease landscape. One CPG [25] was “Not Recommended” because of the lack of clear recommendations, limited applicability information, and lower score on Rigor of Development (e.g., expert consensus without a review of the literature).

## Discussion

In this SLR of international CPGs for DMO, we assessed and compared the methodological rigour and quality of the CPGs using the AGREE II tool, as well as the recommendations of each CPG on the usage of OCT and VA in routine clinical practice. Across all 14 CPGs, we found a strong consensus for recommending OCT and VA in screening, diagnosing, and monitoring DMO. The recommendations, although variable in their applicability, did not vary largely across CPGs, highlighting the ubiquitous nature of these tools in clinical decision-making. However, only two of the CPGs were fully “Recommend” without any reviewer-suggested modifications, based on the AGREE II tool assessments of the CPGs’ quality [30, 31].

VA assessments as a part of the clinical examination have been standard in evaluating DMO [36]. However, OCT is now a routine measure to supplement decision-making [37–39]. As such, we found a strong alignment among geographically global CPGs on the importance of OCT for DMO management from screening to diagnosis and treatment decisions. There is a strong alignment among CPGs that OCT should be used throughout the care continuum, which reinforces the importance of oedema control (i.e., fluid management) in DMO. Lastly, among the CPGs we assessed, none specified the use of one anti-VEGF therapy over another.

In contrast to the management approaches observed in neovascular age-related macular degeneration, physicians typically do not treat DMO on a uniform frequency (i.e. every 8 weeks or every 12 weeks), rather they adhere to a treat-and-extend or pro-re-nata process [40]. This is because if fluid accumulates after treatment for neovascular age-related macular degeneration, it could lead to irreversible vision changes, whereas fluid accumulation does not lead to permanent damage in DMO [41].

Our analysis of the AGREE II domains found that most CPGs were of high quality regarding the Scope and Purpose, Clarity of Recommendations, and Editorial Independence domains, with 13 of 14 CPGs scoring >70% in each of those domains. In contrast, the AGREE II tool also highlighted several areas in which future CPGs in DMO could be improved. For example, Applicability in CPG development was consistently low across most CPGs, and both Stakeholder Involvement (no CPG scored >70%) and Rigor of Development (one CPG scored >70%) criteria were often unmet. Multiple CPGs did not report implementing systematic methods to search for relevant evidence, thereby skipping an essential step that should always precede formulating recommendations [42]. As there were eight items in the Rigor of Development domain, the average score of this one domain had an outsized role in the overall score. We ascertain that the variability found among CPGs is likely because of the lack of standardised study identification upfront (i.e., SLRs were typically not mentioned) and global randomised controlled trials informed most of the recommendations.

More rigorous methods, guidance on applicability in resource-constrained systems, and patient advocacy/perspectives should ideally be incorporated into future CPGs to strengthen their utility for providers. Especially with the burden of treatment in DMO produces challenges in compliance and adherence to treatment, as seen in those with macular degeneration, use of appropriate quality of life instruments could support inclusion of patient perspectives [43]. With multiple comorbid conditions these patient population often have, numerous healthcare visits, could not only be stressful for the patients but also for their caregivers and other healthcare providers who may be accountable for the management of several HCP appointments [43]. Given that the adherence to treatment directly related to treatment outcomes as shown by previous studies, it would be crucial for future CPGs to incorporate patient perspectives such as incorporating treatment satisfaction scores to consider the intensity of treatment when deciding on DMO treatments [44–46]. Additionally, CPG development should consider describing in detail the methodological approach (e.g., methods used for identifying evidence that recommendations are based on, clearly describing the strengths and limitations of the evidence, and forming an explicit link between the CPG recommendations and the supporting evidence).

Importantly, only one CPG mentioned a procedure for updating the current guideline or included the patient preferences/views in the CPG development. Previous research has found that CPGs should ideally be reassessed approximately 3–5 years after publication to ensure the recommendations set forth are still valid [47]. Yet nearly all identified CPGs failed to mention a procedure or time frame for updating. In fact, Amoaku et al. emphasised the formation of the *UK Consensus Working Group* to develop recommendations as the Royal College of Ophthalmologists DR Guidelines are outdated and there is no planned revision [24].

A few limitations should be noted in our SLR. Firstly, the AGREE II tool does not set a threshold for quality, so the reviewers implemented a threshold of <30% as low quality and >70% as high quality across the individual domains. The overall quality and recommendations for use in practice (Recommend, Recommend with Modifications, Do Not Recommend) were also based on the reviewers’ judgement and could be considered subjective to the reviewers’ assessment of each CPG. However, the overall recommendation was made taking into consideration the AGREE II assessment process. Secondly, some of the items in each domain may be out of scope or budget for existing ophthalmology professional groups (e.g., methods for updating the guidelines or establishing monitoring/auditing criteria). Moreover, the focus of this study was on anti-VEGF therapies and the utility of OCT and VA measures in the diagnosis and management of DMO; therefore, we did not capture recommendations on other treatment modalities, such as steroids or laser photocoagulation that may be employed more often in certain regions. As such, this study did not encompass all therapeutic options. Lastly, this study relied on the identification of CPGs via a systematic literature search in general electronic databases (e.g., MEDLINE, Embase). We also performed an extensive grey literature search on Google across organisations and disease-specific websites, which yielded additional CPGs that were not included in our electronic search or published in academic journals. Despite these efforts, we may have inadvertently missed identifying publications if they did not come across our reviewers during the screening period.

In conclusion, our study contributes novel insights into identifying both strengths of CPGs and areas for improvement that should be considered when developing future CPGs for DMO management. Through a thorough and geographically inclusive systematic review and assessment, the study highlights several areas of improvement for future CPGs to ensure high-quality guidelines are available for retina specialist to manage DMO such as improving methodological rigour and increasing stakeholder involvement. Given that DMO is a multifactorial disease state that requires treatment with frequent monitoring and injections, developing a CPGs that is more inclusive of patient perspectives would possibly reduce the treatment burden on patients and optimise disease management by fostering compliance and enhance treatment outcomes for DMO [43, 48].

## Summary

### What was known before

- Unlike other therapeutic areas with authoritative prescribing and disease management guidelines (e.g., National Comprehensive Cancer Network guidelines for oncologic conditions or American College of Rheumatology for immunological conditions), ophthalmology recommendations within clinical practice guidelines (CPGs) are variable, most notably with regard to the latitude that retina specialists are given to base their management decisions. Presently, there is a lack of standardised best practices for the management of DMO. Many retina specialists and ophthalmologists in academic practice are likely more knowledgeable about the most up-to-date literature; however, in more resource-constrained settings, treatment may be more variable, which could lead to potential health disparity issues where the quality of care patients are receiving might be different depending on the care settings.

### What this study adds

- Across all identified guidelines, we found a strong consensus for recommending OCT and VA in screening, diagnosing, and monitoring DMO. The recommendations, although variable in their applicability, did not vary largely across CPGs, highlighting the ubiquitous nature of these tools in clinical decision-making. SLR identified both strengths of CPGs and areas for improvement that should be considered when developing future CPGs for DMO management. Most importantly, improving methodological rigour, guidance on applicability in resource-constrained systems, and patient advocacy/perspectives should ideally be incorporated into future CPGs to ensure high-quality guidelines are available for retina specialists to manage DMO.

## Supplementary information

Supplementary Table Legend

Supplementary Table 1

Supplementary Table 2

## Data Availability

The datasets generated during and/or analysed during the current study are available from the corresponding author on reasonable request.
