# Supplementary material for: Clinical practice guidelines for the treatment and management of diabetic macular oedema: a systematic review
Source: Eye (Lond). 2025 Oct 1;39(17):3121–8. doi: 10.1038/s41433-025-04043-2 (PMC12623899; doi:10.1038/s41433-025-04043-2)
Supplement: Supplementary file 1 — Supplementary Table Legend [file 41433_2025_4043_MOESM1_ESM.docx]

**Supplementary Table Legend**

**Supplementary Table. S1** Detailed Search Strategy.

*Search for additional ophthalmologic conditions conducted in tandem (i.e., retinal vein occlusion, neovascular age-related macular degeneration).

**Supplementary Table. S2** AGREE II Quality Instrument Domains [19]

AGREE II, Appraisal of Guidelines for Research and Evaluation II.
