# Supplementary material for: Clinical practice guidelines for the treatment and management of diabetic macular oedema: a systematic review
Source: Eye (Lond). 2025 Oct 1;39(17):3121–8. doi: 10.1038/s41433-025-04043-2 (PMC12623899; doi:10.1038/s41433-025-04043-2)
Supplement: Supplementary file 2 — Supplementary Table 1 [file 41433_2025_4043_MOESM2_ESM.pdf]

Supplementary Table S1. Detailed search strategy.

#	Search terms (title)	Hits
1	“diabetic macular edema” OR “diabetic macular oedema” OR “diabetic retinopathy” OR “neovascular age-related macular degeneration” OR “wet age-related macular degeneration” OR “neovascular age related macular degeneration” OR “retinal vein occlusion”	88 101*
2	guideline OR “practice guidelines” OR (“practice” AND “guidelines”) OR (“best” AND “practice”) OR “best practice” OR “Preferred Practice Pattern” OR consensus OR “executive summary” OR (executive AND summary) OR “expert consensus” OR “scientific statement” OR “consensus paper” OR “consensus statement”	298 866
3	#1 AND #2	163
4	Publication after 2010; remove duplicates	133

*Search for additional ophthalmologic conditions conducted in tandem (i.e., retinal vein occlusion, neovascular age-related macular degeneration).
