# Supplementary material for: Clinical practice guidelines for the treatment and management of diabetic macular oedema: a systematic review
Source: Eye (Lond). 2025 Oct 1;39(17):3121–8. doi: 10.1038/s41433-025-04043-2 (PMC12623899; doi:10.1038/s41433-025-04043-2)
Supplement: Supplementary file 3 — Supplementary Table 2 [file 41433_2025_4043_MOESM3_ESM.pdf]

Supplementary Table S2. AGREE II Quality Instrument Domains [19]

AGREE II domain	Criteria evaluated
1. Scope and Purpose	• The overall objectives of the guideline are specifically described<br>• The health questions covered by the guideline are specifically described<br>• The population to whom the guideline is meant to apply is specifically described
2. Stakeholder Involvement	• The guideline development group includes individuals from all relevant professional groups<br>• The views and preferences of the target population have been sought<br>• The target users of the guideline are clearly defined
3. Rigor of Development	• Systematic methods were used to search for evidence<br>• The criteria for selecting the evidence are clearly described<br>• The strengths and limitations of the body of evidence are clearly described<br>• The methods for formulating the recommendations are clearly described<br>• The health benefits, side effects and risks have been considered in formulating the recommendations<br>• There is an explicit link between the recommendations and the supporting evidence<br>• The guideline has been externally reviewed by experts prior to publication<br>• A procedure for updating the guideline is provided
4. Clarity of Presentation	• The recommendations are specific and unambiguous<br>• The different options for management of the condition or health issue are clearly presented<br>• Key recommendations are easily identifiable

5. Applicability	• The guideline describes facilitators and barriers to its application<br>• The guideline provides advice and/or tools on how the recommendations can be put into practice<br>• The potential resource implications of applying the recommendations have been considered<br>• The guideline presents monitoring and/or auditing criteria
6. Editorial Independence	• The views of the funding body have not influenced the content of the guideline<br>• Competing interests of guideline development group members have been recorded and addressed

AGREE II, Appraisal of Guidelines for Research and Evaluation II.
